# Supplementary material for: Methylation content sensitive enzyme ddRAD (MCSeEd): a reference-free, whole genome profiling system to address cytosine/adenine methylation changes
Source: Sci Rep. 2019 Oct 16;9:14864. doi: 10.1038/s41598-019-51423-2 (PMC6795852; doi:10.1038/s41598-019-51423-2)
Supplement: Supplementary file 1 — Supplementary Figures [file 41598_2019_51423_MOESM1_ESM.pdf]

Methylation content sensitive enzyme ddRAD (MCSeEd): a reference-free, whole genome profiling system to address cytosine/ adenine methylation changes

Gianpiero Marconi[1], Stefano Capomaccio[2], Cinzia Comino[3], Alberto Acquadro[3], Ezio Portis[3], Andrea Porceddu[4], Emidio Albertini[1]*

[1] Department Agricultural, Food and Environmental Sciences, University of Perugia, Perugia (Italy)

[2] Department of Veterinary Medicine, University of Perugia, Perugia (Italy)

[3] Department of Agricultural, Forest and Food Sciences, Plant Genetics and Breeding, University of Torino, Grugliasco (Italy)

[4] Department of Agriculture, University of Sassari, Sassari (Italy)

***Corresponding author: Emidio Albertini**

Department Agricultural, Food and Environmental Sciences

University of Perugia

Perugia, Italy

Tel: +39/0755856206

Email: emidio.albertini@unipg.it

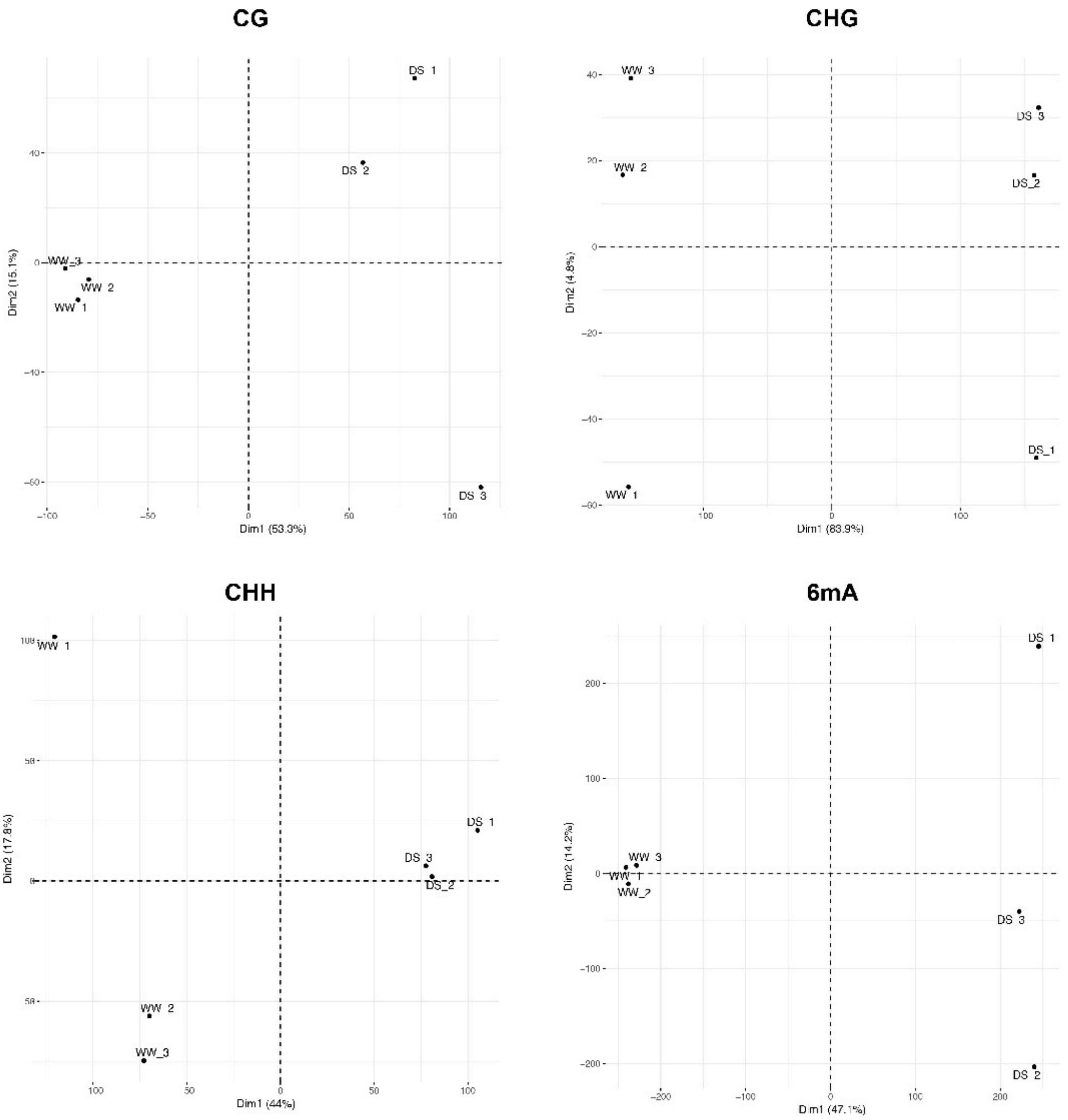

Supplementary Figure S1. Principal component analysis (genome dependent) for the relative methylation levels at the differentially methylated positions obtained across all of the replicates in each sequence context: (a) CG; (b) CHG; (c) CHH; (d) 6mA. Numbers in brackets indicate the fraction of overall variance explained by the respective component (Dim1, Dim2).

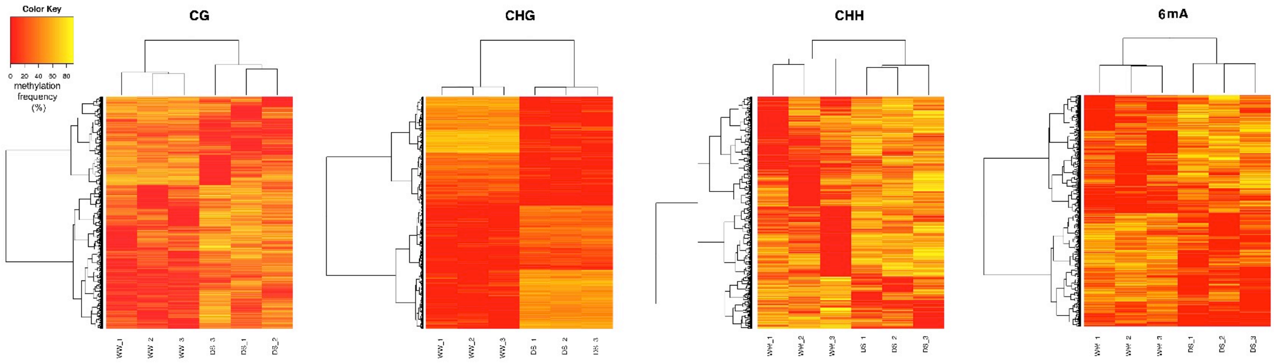

Supplementary Figure S2. Complete linkage clustering based on the relative methylation levels at the differentially methylated positions, as retrieved from the genome-dependent strategy.

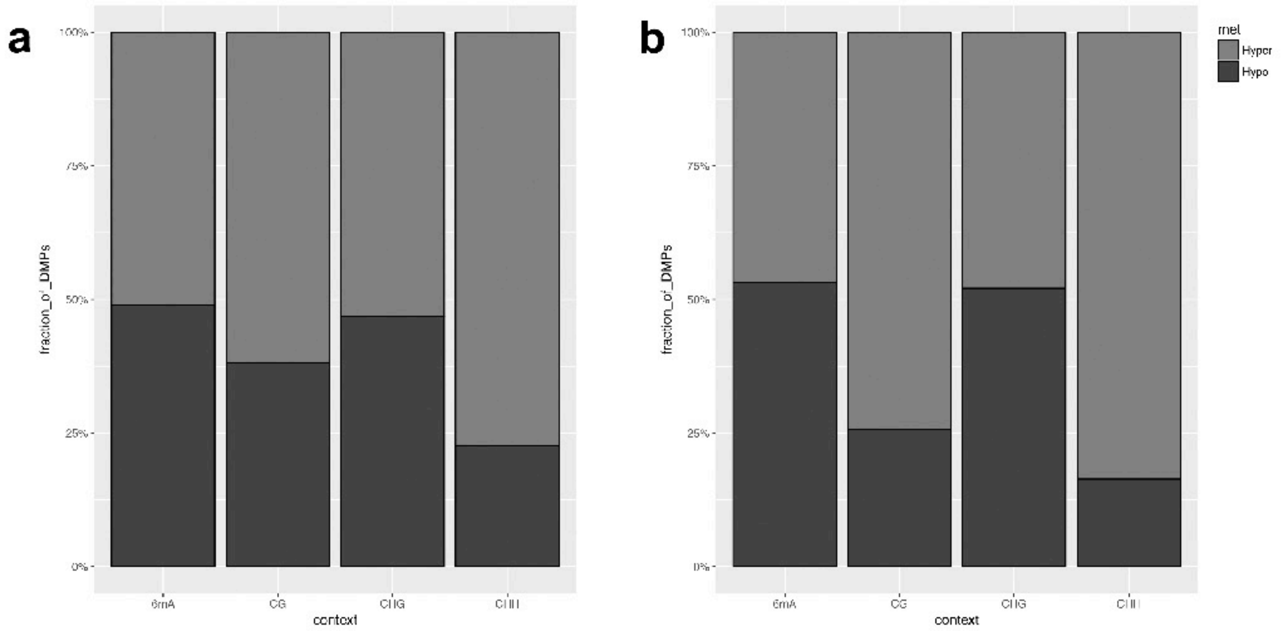

Supplementary Figure S3. Effects of drought stress on the global methylation: (a) genome dependent; (b) genome independent.

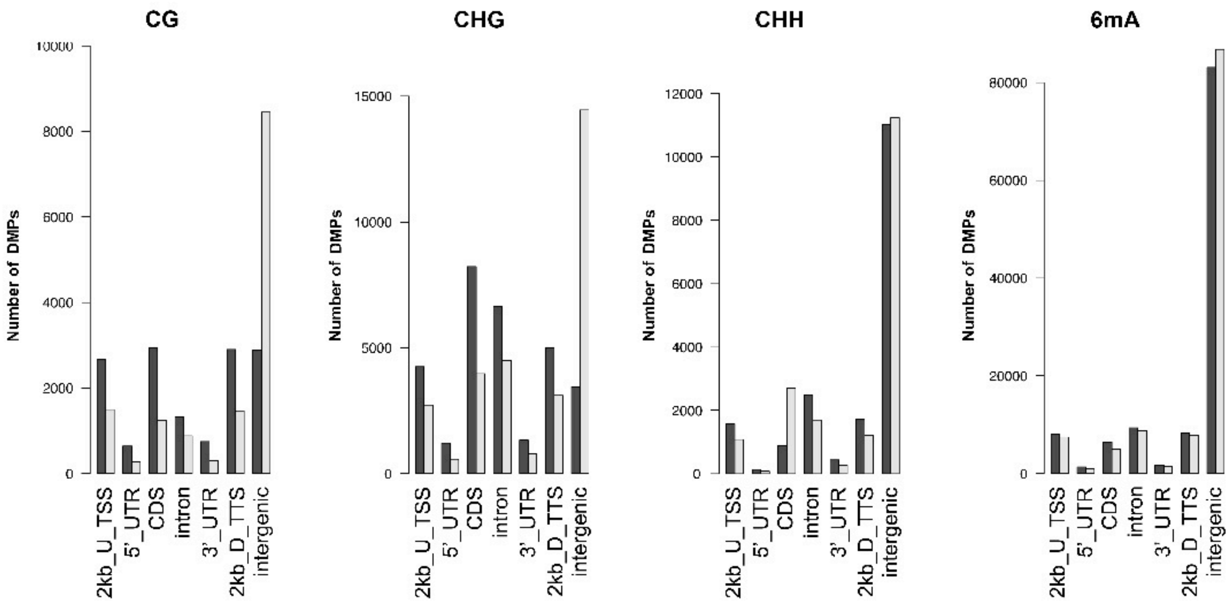

Supplementary Figure S4. Enrichment analyses of the differentially methylated positions in the genomic regions. Enrichment analysis was performed using binomial distribution for all of the MCSeEd loci as expected, and the differentially methylated positions for the CG, CHG, CHH, and 6mA contexts, as the observed datasets. U, upstream; D, downstream. Light gray = expected number of DMPs, Dark gray = observed number of DMPs.

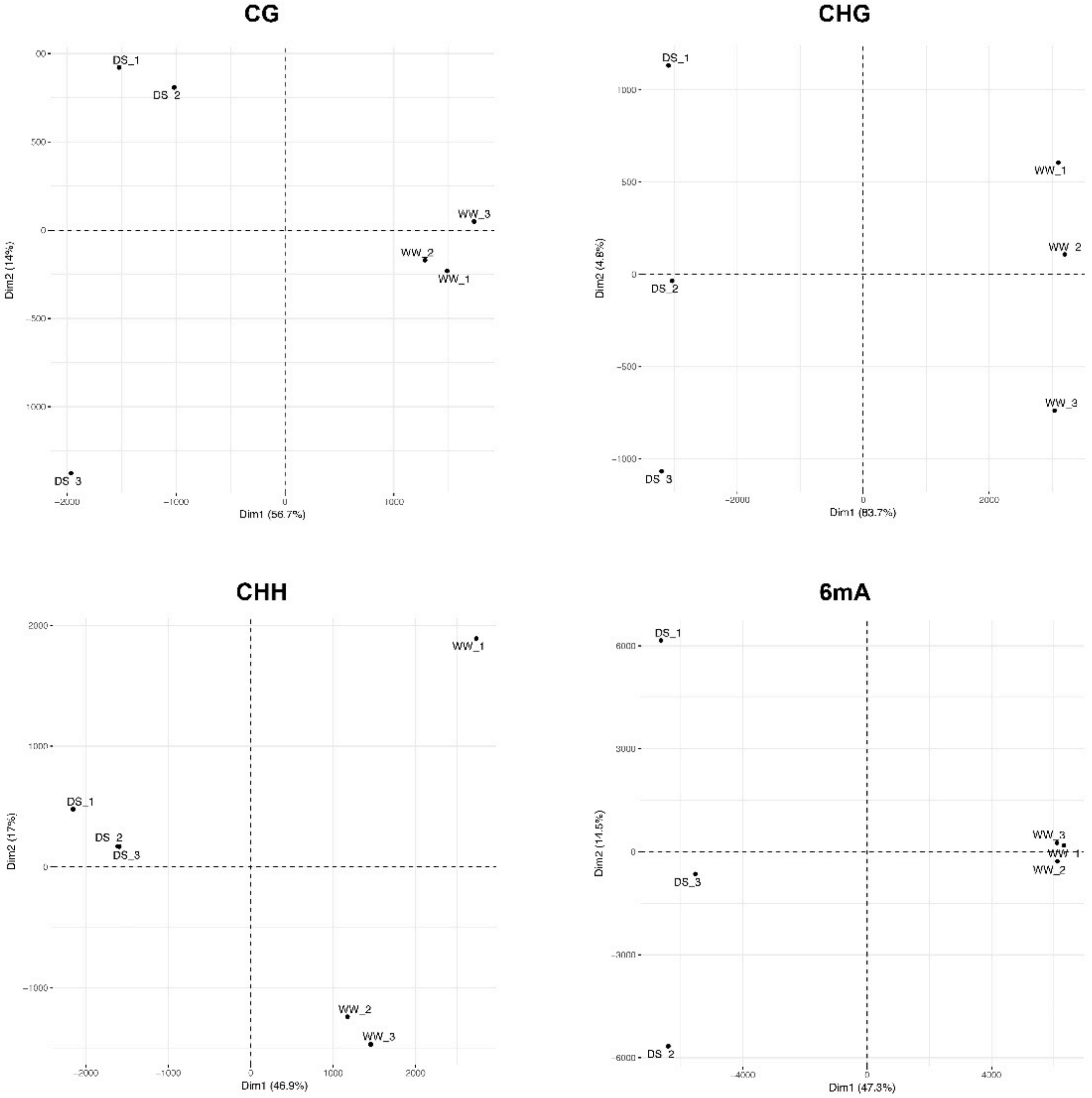

Supplementary Figure S5. Principal component analysis (genome independent) of the relative methylation levels at the differentially methylated positions obtained across all of the replicates in each sequence context: (a) CG; (b) CHG; (c) CHH; (d) 6mA. Numbers in brackets indicate the fraction of overall variance explained by the respective component (Dim1, Dim2).

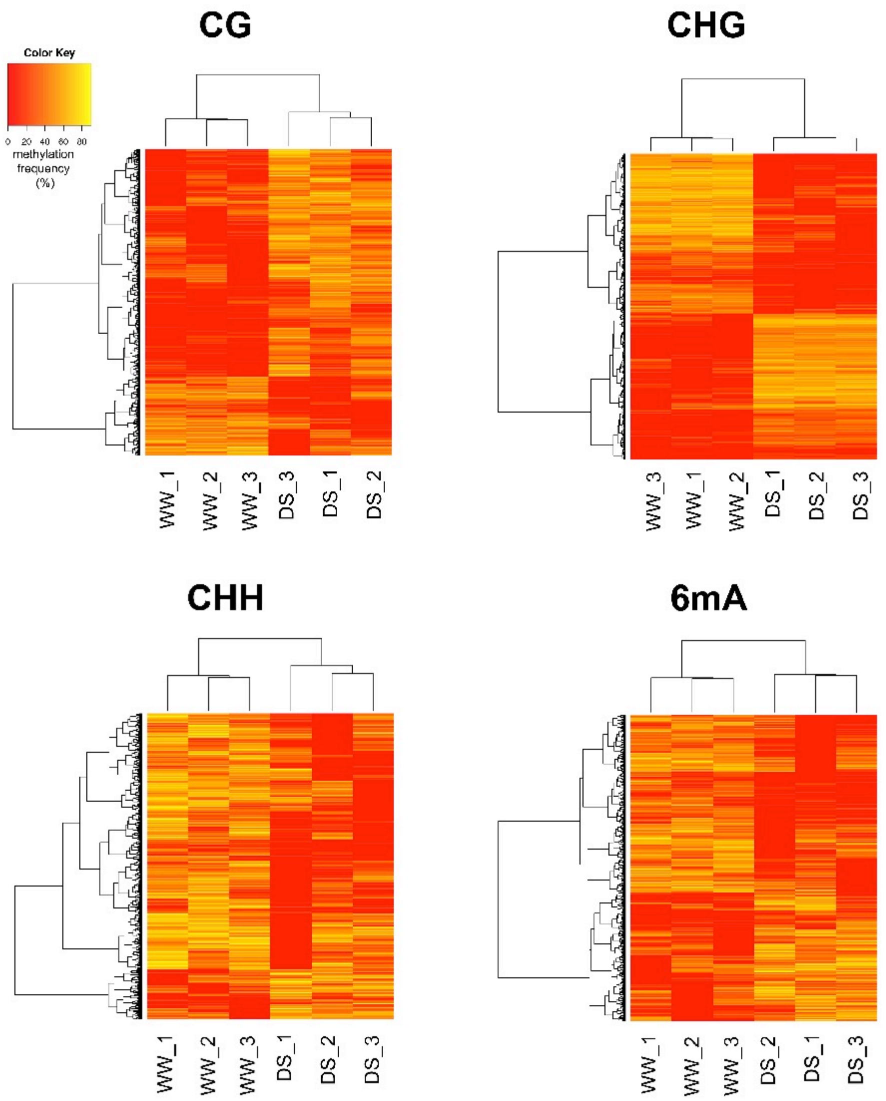

Supplementary Figure S6. Complete linkage clustering based on the relative methylation levels at the differentially methylated positions, as retrieved from the genome-independent strategy.

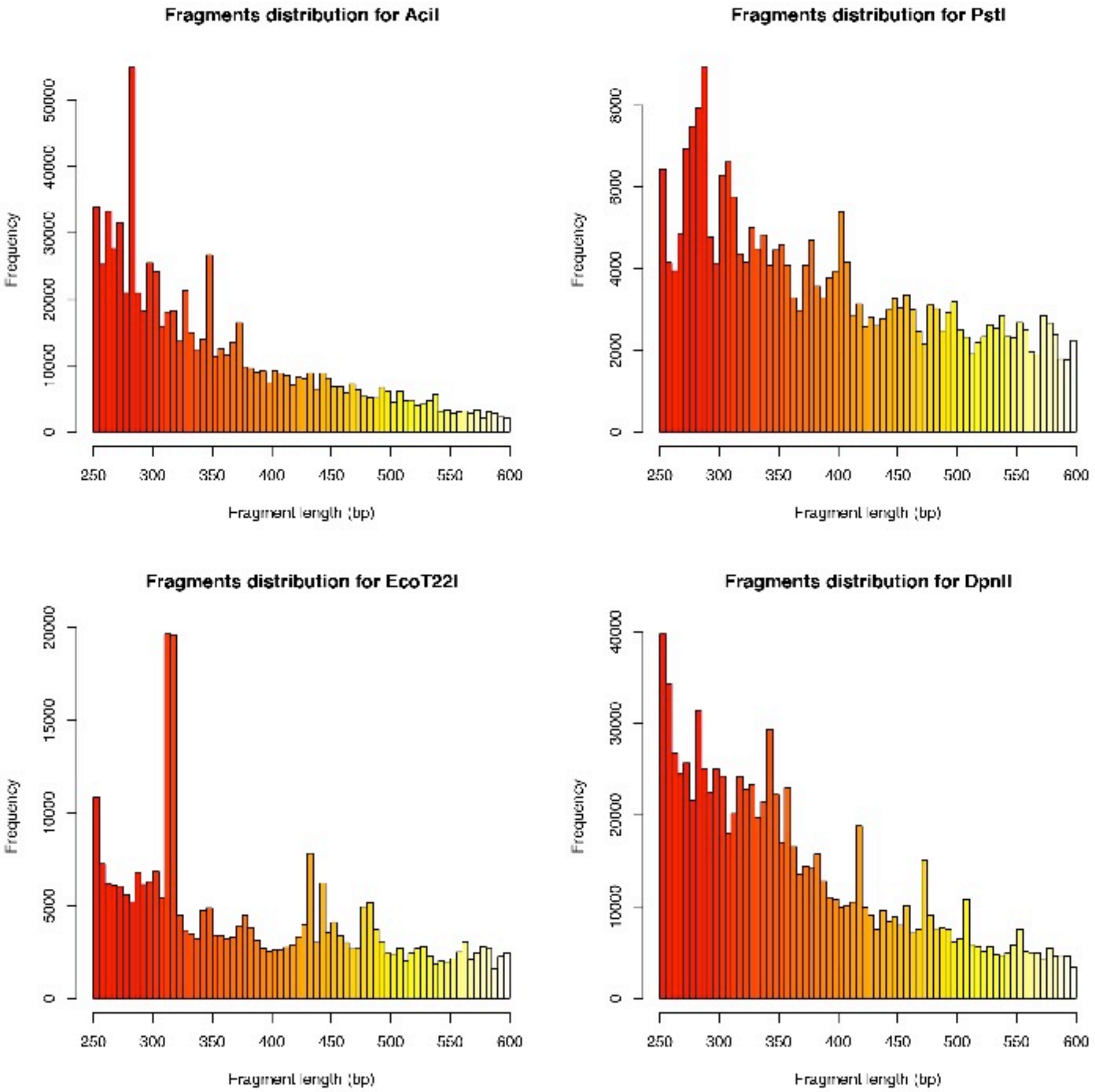

Supplementary Figure S7. Size distribution of restriction fragments for the enzyme combinations used in the study.
